# Risk of intestinal involvement in mucocutaneous-onset Behçet’s disease: data from the AIDA network registry

**DOI:** 10.3389/fimmu.2026.1778171

**Published:** 2026-05-13

**Authors:** Antonio Vitale, Valeria Caggiano, Jessica Sbalchiero, Francesco Gavioli, Giuseppe Lopalco, Gaafar Ragab, Silvana Guerriero, Ibrahim Almaglouth, Abdurrahman Tufan, Roberto Giacomelli, Haner Direskeneli, Piero Ruscitti, Gülen Hatemi, Francesco Carubbi, Ezgi Deniz Batu, Seza Ozen, Jurgen Sota, Henrique Ayres Mayrink Giardini, Micol Frassi, Petros P Sfikakis, Maria Morrone, Federica Gatti, Amina Maher, Ayman Abdel-Monem Ahmed Mahmoud, Rosanna Dammacco, Hamit Kucuk, Riza Can Kardas, Ibrahim Yahya Cakir, Letizia Pia di Corcia, Fatma Alibaz Öner, Gizem Sevik, Martina Gentile, Alican Karakoc, Alessia Alunno, Hulya Ercan Emreol, Francesca Crisafulli, Maria Tektonidou, Francesco Ciccia, Maissa Thabet, Serena Bugatti, Alessandra Milanesi, Maria Sole Chimenti, Benedetta Monosi, Matteo Piga, Alberto Floris, Andrea Hinojosa-Azaola, Guillermo Arturo Guaracha-Basañez, Cecilia Beatrice Chighizola, José Hernández-Rodríguez, Marco Cattalini, Marcello Govoni, Ombretta Viapiana, Adele Civino, Daniela Opris-Belinski, Carla Gaggiano, Rosaria Talarico, Annarita Giardina, Giacomo Emmi, Piercarlo Sarzi Puttini, Maria Cristina Maggio, Paola Parronchi, Piero Portincasa, Alejandra de-la-Torre, Juanita Cardona-López, Stefano Gentileschi, Angela Mauro, Gian Domenico Sebastiani, Maria Francesca Gicchino, Ali Şahin, Donato Rigante, Emre Bilgin, Emanuela Del Giudice, Luciana Breda, Amato De Paulis, Alberto Lo Gullo, Şükran Erten, Samar Tharwat, Lampros Fotis, Maier Armin, Antonella Insalaco, Anastasios Karamanakos, Alessandro Conforti, Özgül Soysal Gündüz, Abdelhfeez Moshrif, Francesca Li Gobbi, Stefania Costi, Elena Bartoloni, Patrizia Barone, Serena Guiducci, Andrés González-García, Inés Hernanz Rodriguez, Giovanni Conti, Annamaria Iagnocco, Fatos Önen, Sulaiman M. Al-Mayouf, Ali İbrahim Hatemi, Alberto Balistreri, Claudia Fabiani, Luca Cantarini

**Affiliations:** 1 Rheumatology Unit, Department of Medical Sciences, Surgery and Neurosciences Department of Medical Sciences, Surgery and Neurosciences, University of Siena and Azienda Ospedaliero-Universitaria Senese, Siena, Italy; 2 Department of Precision and Regenerative Medicine and Ionian Area (DiMePRe-J) Policlinic Hospital, University of Bari, Bari, Italy; 3 Internal Medicine Department, Rheumatology and Clinical Immunology Unit, Faculty of Medicine, Cairo University, Giza, Egypt; 4 Faculty of Medicine, Newgiza University, 6th of October, Giza, Egypt; 5 Department of of Translational Biomedicine and Neuroscience (DiBraiN), University of Bari, Bari, Italy; 6 Rheumatology Unit, Department of Medicine, King Saud University, Riyadh, Saudi Arabia; 7 Division of Rheumatology, Department of Internal Medicine, Gazi University Hospital, Ankara, Türkiye; 8 Rheumatology, Immunology and Clinical Medicine Unit, Department of Medicine, Università Campus Bio-Medico di Roma, Rome, Italy; 9 Department of Internal Medicine, Division of Rheumatology, School of Medicine, Marmara University, Istanbul, Türkiye; 10 Rheumatology Unit, Department of Biotechnological and Applied Clinical Sciences, University of L'Aquila, L'Aquila, Italy; 11 Department of Internal Medicine, Division of Rheumatology, Cerrahpasa Medical School, Istanbul University-Cerrahpasa, Istanbul, Türkiye; 12 University of L'Aquila, Department of Life, Health & Environmental Sciences, Internal Medicine and Nephrology Division, ASL1 Avezzano-Sulmona-L'Aquila, San Salvatore Hospital, L'Aquila, Italy; 13 Pediatric Rheumatology Unit, Department of Pediatrics, Hacettepe University School of Medicine, Ankara, Türkiye; 14 Rheumatology Division, Faculdade de Medicina, Hospital das Clinicas (HCFMUSP), Universidade de Sao Paulo, Sao Paulo, Brazil; 15 Rheumatology and Clinical Immunology, Spedali Civili and Department of Clinical and Experimental Sciences, University of Brescia, Brescia, Italy; 16 Joint Academic Rheumatology Program, National and Kapodistrian University of Athens Medical School, Athens, Greece; 17 Department of Precision Medicine, Università Degli Studi Della Campania Luigi Vanvitelli, Naples, Italy; 18 Internal Medicine Department, Farhat Hached University Hospital, Faculty of Medicine of Sousse, University of Sousse, Sousse, Tunisia; 19 Department of Internal Medicine and Therapeutics, Università di Pavia and Division of Rheumatology, Fondazione Istituto di ricovero ecura a carattere scientifico (IRCCS) Policlinico San Matteo, Pavia, Italy; 20 Rheumatology, Allergology and Clinical Immunology, Department of Systems Medicine, University of Rome Tor Vergata, Rome, Italy; 21 Rheumatology Unit, Department of Medical Sciences, University and AOU of Cagliari, Cagliari, Italy; 22 Department of Immunology and Rheumatology, Instituto Nacional de Ciencias Médicas Y Nutrición Salvador Zubirán, Mexico City, Mexico; 23 Pediatric Rheumatology Unit, Azienda Socio-Sanitaria Territoriale (ASST) Gaetano Pini Centro Specialistico Ortopedico Traumatologico (CTO), Milan, Italy; 24 Department of Clinical Sciences and Community Health, Research Center for Adult and Pediatric Rheumatic Diseases, University of Milan, Milan, Italy; 25 Autoinflammatory Diseases Clinical Unit, Department of Autoimmune Diseases, Hospital Clinic of Barcelona, August Pi I Sunyer Biomedical Research Institute (IDIBAPS), University of Barcelona, Barcelona, Spain; 26 Pediatric Clinic, University of Brescia and Spedali Civili di Brescia, Brescia, Italy; 27 Rheumatology Unit, Department of Medical Sciences, Azienda Ospedaliero-Universitaria S. Anna - Ferrara, University of Ferrara, Ferrara, Italy; 28 Rheumatology Unit, Department of Medicine, University and Azienda Ospedaliera Universitaria Integrata of Verona, Verona, Italy; 29 Pediatric Rheumatology and Immunology Unit, Vito Fazzi Hospital, Lecce, Italy; 30 Rheumatology and Internal Medicine Department, Carol Davila University of Medicine and Pharmacy, Bucharest, Romania; 31 Rheumatology Unit, Department of Clinical and Experimental Medicine, University of Pisa, Pisa, Italy; 32 UOC Medicina Interna, Ambulatorio di Reumatologia, Azienda ospedaliera di rilievo nazionale e di alta specializzazione (ARNAS) Civico Di Cristina Benfratelli, Palermo, Italy; 33 Department of Medical, Surgical and Health Sciences, and Clinical Medicine and Rheumatology Unit, Cattinara University Hospital, University of Trieste, Trieste, Italy; 34 Centre for Inflammatory Diseases, Department of Medicine, Monash Medical Centre, Monash University, Clayton, VIC, Australia; 35 Rheumatology Unit, Ospedale Sacco, Milan, Italy; 36 University Department of Health Promotion, Mother and Child Care, Internal Medicine and Medical Specialties (PROMISE) "G. D'Alessandro", University of Palermo, Palermo, Italy; 37 Department of Experimental and Clinical Medicine, University of Florence, Florence, Italy; 38 Clinica Medica "A. Murri", Division of Internal Medicine, Department of Precision and Regenerative Medicine and Ionian Area (DiMePre-J), University of Bari Aldo Moro, Bari, Italy; 39 Neuroscience Research Group (NEUROS), Neurovitae Center for Neuroscience, Institute of Translational Medicine (IMT), School of Medicine and Health Sciences, Universidad del Rosario, Bogotá, Colombia; 40 Department of Biomedical and Clinical Sciences, Fatebenefratelli Hospital, Università di Milano, Milan, Italy; 41 Pediatric Rheumatology Unit, Department of Childhood and Developmental Medicine, Fatebenefratelli-Sacco Hospital, Milan, Italy; 42 Unità operativa complessa (U.O.C.) Reumatologia, Ospedale San Camillo-Forlanini, Rome, Italy; 43 Department of Woman, Child and of General and Specialized Surgery, University of Campania "Luigi Vanvitelli", Naples, Italy; 44 Division of Rheumatology, Department of Internal Medicine, Sivas Cumhuriyet University Medical Faculty, Sivas, Türkiye; 45 Department of Life Sciences and Public Health, Fondazione Policlinico Universitario A. Gemelli Istituto di ricovero e cura a carattere scientifico (IRCCS), Rome, Italy; 46 Rare Diseases and Periodic Fevers Research Centre, Università Cattolica Sacro Cuore, Rome, Italy; 47 Division of Rheumatology, Faculty of Medicine, Sakarya University, Sakarya, Türkiye; 48 Department of Maternal Infantile and Urological Sciences, Sapienza University of Rome, Polo Pontino, Rome, Italy; 49 Department of Paediatrics, University of Chieti-Pescara, Chieti, Italy; 50 Department of Translational Medical Sciences, Section of Clinical Immunology, University of Naples Federico II, Naples, Italy; 51 Center for Basic and Clinical Immunology Research (CISI), WAO Center of Excellence, University of Naples Federico II, Naples, Italy; 52 Unit of Rheumatology, Department of Medicine, Azienda ospedaliera di rilievo nazionale e di alta specializzazione (ARNAS) Garibaldi Hospital, Catania, Italy; 53 Department of Rheumatology, Faculty of Medicine Ankara City Hospital, Ankara Yildirim Beyazit Universitesi, Ankara, Türkiye; 54 Rheumatology and Immunology Unit, Internal Medicine Department, Mansoura University, Mansoura, Egypt; 55 Department of Internal Medicine, Faculty of Medicine, Horus University, New Damietta, Egypt; 56 Department of Pediatrics, Attikon General Hospital, National and Kapodistrian University of Athens, Athens, Greece; 57 Rheumatology Unit, Department of Medicine, Central Hospital of Bolzano, Bolzano, Italy; 58 Division of Rheumatology, Ospedale Pediatrico Bambino Gesù, Istituto di ricovero ecura a carattere scientifico (IRCCS), Rome, Italy; 59 Department of Rheumatology, "Evangelismos" General Hospital, Athens, Greece; 60 Ospedale San Paolo di Civitavecchia, U.O. Medicina Generale, ASL Roma 4, Rome, Italy; 61 Division of Rheumatology, Department of Internal Medicine, School of Medicine, Manisa Celal Bayar University, Manisa, Türkiye; 62 Rheumatology Department, Faculty of Medicine, Al-Azhar University, Assiut, Egypt; 63 Rheumatology Unit, Hospital S. Giovanni di Dio, Azienda USL-Toscana Centro, Florence, Italy; 64 Rheumatology Unit, Department of Medicine and Surgery, University of Perugia, Perugia, Italy; 65 Department of Clinical and Experimental Medicine, University of Catania, Catania, Italy; 66 Division of Rheumatology, Department of Experimental and Clinical Medicine, University of Florence, Florence, Italy; 67 Systemic Autoimmune Diseases Unit, Department of Internal Medicine, Hospital Universitario Ramón y Cajal, Istituto Ramon y Cajal de Investigation Sanitaria (IRYCIS), Madrid, Spain; 68 Department of Ophthalmology, Hospital Universitario Fundación Jiménez Díaz, Madrid, Spain; 69 Pediatric Nephrology and Rheumatology Unit, Azienda Ospedaliero Universitaria (AOU) G Martino, Messina, Italy; 70 Academic Rheumatology Center, Dipartimento Scienze Cliniche e Biologiche, Università degli Studi di Torino, Turin, Italy; 71 Department of Internal Medicine, Division of Rheumatology, School of Medicine, Dokuz Eylül University, Izmir, Türkiye; 72 Department of Pediatrics, King Faisal Specialist Hospital and Research Center, College of Medicine, Alfaisal University, Riyadh, Saudi Arabia; 73 Department of Internal Medicine, Division of Gastroenterology, Cerrahpasa Medical School, Istanbul University-Cerrahpasa, Istanbul, Instabul, Türkiye; 74 Bioengineering and Biomedical Data Science Lab, Department of Medical Biotechnologies, University of Siena, Siena, Italy; 75 Ophthalmology Unit, Department of Medicine, Surgery and Neurosciences, University of Siena and Azienda Ospedaliero-Universitaria Senese, Siena, Italy

**Keywords:** gut, inflammation, precision medicine, predictors, risk factors

## Abstract

**Objective:**

Behçet’s disease (BD) may initially manifest solely with mucocutaneous involvement. This study aimed to identify demographic and clinical factors associated with subsequent intestinal involvement in patients presenting exclusively with mucocutaneous manifestations during early disease stages.

**Methods:**

Data were obtained from the International AutoInflammatory Disease Alliance Network registry dedicated to BD; a Bayesian statistical approach was employed to address the limited sample size resulting from subgroup stratifications.

**Results:**

In total, 328 BD patients with exclusively mucocutaneous onset were enrolled; of these, 46 (14%) developed intestinal involvement over time. The risk of ocular involvement was higher among patients with intestinal manifestations (OR: 3.02, 95% CrI: 1.24–6.08; posterior probability: 99.3%). Minor aphthous ulcers without major aphthosis were protective towards intestinal involvement (OR: 0.47, 95% CrI: 0.22–0.98; posterior probability: 97.98%). Conversely, major aphthous ulcers increased the risk (OR: 3.25, 95% CrI: 1.50–7.02; posterior probability: 99.7%), along with the combination of oral major aphthosis with: i) genital aphthosis (OR = 2.77, 95%CrI: 1.14-6.58; posterior probability: 98.45%); ii) pseudofolliculitis (OR = 3.18, 95%CrI: 1.15-8.33; posterior probability: 98.72%); iii) genital aphthosis plus pseudofolliculitis (OR = 5.22, 95%CrI: 1.71-16.35; posterior probability of 99.77%). Pseudofolliculitis plus cutaneous manifestations other than erythema nodosum were protective against intestinal involvement (OR = 0.01, 95%CrI: 0.0-0.98; posterior probability: 97.55%).

**Conclusion:**

Major aphthosis was the strongest factor associated with intestinal involvement in BD patients initially presenting with mucocutaneous symptoms only. In such patients, intestinal involvement correlated with increased risk of ocular inflammation.

## Highlights

- Major oral aphthosis is the strongest predictor of subsequent intestinal involvement in Behçet’s disease patients who initially present with only mucocutaneous symptoms.
- Specific combinations of mucocutaneous features, particularly major oral aphthosis with genital aphthosis and/or pseudofolliculitis, significantly increase the risk of intestinal manifestations, while minor aphthosis appears protective.
- Intestinal involvement is linked to a higher likelihood of ocular inflammation, suggesting overlapping pathogenic mechanisms and highlighting the need for careful multidisciplinary monitoring in high-risk patients.

## Introduction

Behçet’s syndrome (BD) is a multifactorial disease characterised by a heterogeneous spectrum of clinical features including mucocutaneous, articular, ocular, vascular, neurological, and gastrointestinal manifestations (1). However, BD may initially present clinical manifestations limited to mucocutaneous involvement, including recurrent oral and genital ulcers, pseudofolliculitis, erythema nodosum, skin ulcers, pyoderma gangrenosum, and other polymorphic cutaneous eruptions (2). Nevertheless, the disease may later evolve to major organ involvement, potentially affecting the eye, the vascular system, the central nervous system (CNS), and the gastrointestinal tract (3, 4). Intestinal involvement may manifest with features closely resembling inflammatory bowel diseases, particularly Crohn’s disease (5). Typical lesions include deep, well-demarcated ulcers, most commonly located in the terminal ileum and cecum, although any segment of the gastrointestinal tract may be affected (6, 7). Abdominal pain and diarrhoea represent the most common clinical symptoms, complicated by intestinal perforation or massive hemorrhage in severe cases (1). Intestinal involvement is often associated with greater BD severity and can be life-threatening, thus requiring the use of immunosuppressive or biotechnological therapies (8). This progression highlights the importance of regular clinical monitoring even in patients initially presenting with an apparently benign phenotype. In this regard, it is useful to identify clinical features that may help distinguish, among patients presenting mucocutaneous involvement only, those who are more likely to develop intestinal involvement. This has important practical implications for assessing the risk of developing a potentially disabling and life-threatening complication, which warrants closer follow-up and an appropriate therapeutic approach. Therefore, the present study was conducted to identify clinical characteristics associated with an increased risk of developing intestinal involvement in patients presenting mucocutaneous manifestations as the sole form of BD in the early stages.

## Materials and methods

Demographic, genetic and clinical data were obtained from the International AutoInflammatory Disease Alliance (AIDA) Network registry dedicated to BD (9). All participants fulfilled the classification criteria established by the International Study Group (ISG) and/or the International Criteria for BD (ICBD) (10, 11).

For this analysis, patients whose initial disease presentation was restricted exclusively to mucocutaneous involvement were selected. Specifically, eligible patients presented with oral aphthosis, genital ulcers, and/or BD-related cutaneous lesions, without any evidence of ocular, gastrointestinal, vascular, or CNS involvement for at least 12 months following disease onset. Disease onset was defined as the time point at which the patient first fulfilled the established classification criteria. Intestinal involvement was defined as the presence of inflammation and/or ulceration within the gastrointestinal tract, with occurrence of abdominal pain, persistent diarrhea, hematochezia, intestinal occlusion or stenosis, or fistulas. In this context, recurrent diarrhea and abdominal pain were considered relevant only when accompanied by a significant elevation in faecal calprotectin levels (≥130 mg/kg). Guided by clinical necessity, an endoscopy was performed to identify any macroscopic lesions or histological alterations compatible with intestinal inflammatory involvement associated with BD. Alternative causes of intestinal inflammation or ulceration were meticulously ruled out through targeted investigations tailored to each individual clinical presentation.

Patients were subsequently stratified according to whether they developed gastrointestinal involvement over the course of the disease. A comprehensive assessment was conducted, including demographic and clinical variables, human leukocyte antigen (HLA)-B51 status, the size and number of concomitant oral and genital ulcers, as well as the full spectrum of BD-related cutaneous manifestations. BD activity at the start of the disease was evaluated using the transformed BD current activity form (BDCAF), if available within two years from the disease onset (12).

Oral and genital ulcer count at disease onset was categorized into three groups: 1–2 lesions, 3–5 lesions, and more than 5 lesions. Based on size-based classifications of oral ulcers proposed in the literature (13), oral aphthae were further classified into minor (<10 mm in diameter), major (>10 mm), and herpetiform (numerous, small, and clustered lesions).

Statistical analysis included descriptive methods such as mean, median, standard deviation (SD), interquartile range, frequency counts, and corresponding percentages. A Bayesian logistic regression framework was employed to assess the association between clinical and demographic variables and the development of intestinal involvement over the course of the disease. The presence or absence of intestinal involvement was modeled as the dependent variable, while individual demographic, clinical, and mucocutaneous features were entered one at a time as independent variables. Models were fitted using the *brms* package in R Studio software, which interfaces with Stan for Bayesian inference via Hamiltonian Monte Carlo. Each model estimated the posterior distribution of the log-odds of intestinal involvement as a function of the selected covariate. A weakly informative normal prior (mean = 0, SD = 5) was applied to all regression coefficients to regularize estimation while avoiding strong assumptions. Each model was run using four Markov chains, each with 2,000 iterations (including 1,000 warm-up and 1,000 sampling steps), ensuring proper convergence and sufficient exploration of the posterior distribution. Posterior summaries were reported as means and 95% credible intervals (CrIs). In addition, the posterior probability of a positive (or negative) association was computed directly from the posterior draws, allowing for intuitive probabilistic interpretation of effect estimates. Odds ratios (ORs) and their corresponding 95% CrIs were obtained by exponentiating the posterior distributions of the regression coefficients. Associations were considered statistically significant when the 95% CrI excluded the null value (OR = 1), and/or when the posterior probability exceeded 97.5%. Variables with posterior probabilities above 95% were considered to show a trend toward significance. All models were adjusted for potential confounders, including maximum disease duration (meant as the time between disease onset and the last visit collected in the AIDA registry), use of conventional disease-modifying antirheumatic drugs (cDMARDs), and anti-tumor necrosis factor (TNF) biotechnological agents.

For all variables that were either statistically significant or showed a trend toward, marginal effects were calculated to quantify the absolute change in the predicted probability of intestinal involvement associated with the presence or absence of the given characteristic.

This Bayesian approach was favored over frequentist methods to provide more informative, flexible, and probabilistically interpretable estimates, particularly given the modest sample size and subgroup stratifications.

When evaluating the effect of combinations of mucocutaneous variables on intestinal involvement using Bayesian logistic regression models, only associations observed in at least 10 patients were considered to ensure statistical stability.

## Results

A total of 328 BD patients with exclusively mucocutaneous involvement at the disease onset were enrolled; of these, 46 (14%) developed intestinal inflammatory involvement over time, as better described in **Table 1**. Among patients developing intestinal involvement over time, 10 (21.7%) also exhibited ocular inflammation, 3 (6.5%) developed vascular involvement and 1 (2.2%) CNS involvement. Bayesian analysis yielded an OR of 3.02 (95% CrI: 1.24-6.08) for the association between ocular and intestinal involvement in BD, with a posterior probability of 99.3% that ocular manifestations are more frequent among patients developing intestinal involvement. In contrast, no substantial evidence of an association was found between intestinal and vascular involvement (OR: 0.63, 95% CrI0. 2-2.8, posterior probability: 74.9%), and between intestinal and CNS involvement (OR: 1.36, 95% CrI 0.14-4.44, posterior probability: 52.1%).

**Table 1:** Demographic and clinical characteristics of the 328 patients with Behçet’s disease who initially presented with only mucocutaneous manifestations, and the type of gastrointestinal involvement subsequently reported in 46 patients during later stages of the disease.

Patients’features	Patients without gut involvement (n=282)	Patients later developing gut involvement (n=46)
Sex (F/M)	149/133	33/13
Country of origin		
Brazil, n (%)	7 (2.5)	0 (0)
Egypt, n (%)	32 (11.4)	3 (6.5)
Greece, n (%)	7 (2.5)	1 (2.2)
Italy, n (%)	160 (56.7)	39 (84.8)
Libya, n (%)	1 (0.4)	0 (0)
Mexico, n (%)	2 (0.7)	1 (2.2)
Romania, n (%)	3 (1.1)	0 (0)
Saudi Arabia, n (%)	17 (6)	1 (2.2)
Spain, n (%)	2 (0.7)	1 (2.2)
Tunisia, n (%)	5 (1.8)	0 (0)
Turkey, n (%)	44 (15.6)	0 (0)
Yemen, n (%)	2 (0.7)	0 (0)
Ethnicity#		
Caucasian, n (%)	193 (68.44%)	40 (86.95%)
Arab, n (%)	55 (19.50%)	5 (10.87%)
Hispanic, n (%)	5 (1.77%)	1 (2.17%)
Other, n (%)	7 (2.48%)	0 (0.0%)
Age at disease onset, years (mean ± SD)	29.53 ( ± 13.99)	28.76 ( ± 14.42)
Age at disease diagnosis, years (mean ± SD)	34.73 ( ± 13.96)	35.21 ( ± 13.56)
HLA-B51 positivity, n (%)*	128 (45.40%)	29 (63.04%)
Positive family history, n (%)	29 (10.28%)	6 (13.04%)
Initial BDCAF value, mean (IQR)	5 (4)	7 (3)
Oral aphthosis features at baseline^§^		
Concurrent oral ulcers (1–2 lesions)	68 (24.11%)	6 (13.04%)
Concurrent oral ulcers (3-5)	82 (29.07%)	19 (41.3%)
Concurrent oral ulcers (>5)	40 (14.18%)	7 (15.21%)
Oral Minor aphthous ulcerations (<10mm)	143 (50.71%)	16 (34.78%)
Oral Major aphthous ulcerations (>10mm)	48 (17.02%)	18 (39.13%)
Oral herpetiform ulcerations	8 (2.83%)	2 (4.34%)
Genital aphthosis features at baseline		
Genital aphthosis	187 (66.31%)	33 (71.74%)
Concurrent genital ulcers (1–2 lesions)^§§^	97 (34.40%)	15 (32.6%)
Concurrent genital ulcers (3–5 lesions) ^§§^	27 (9.57%)	5 (10.87%)
Concurrent genital ulcers (>5 lesions) ^§§^	8 (2.83%)	0 (0.0%)
Skin manifestations at baseline		
Skin involvement	161 (57.09%)	31 (67.39%)
Pseudofolliculitis	117 (41.49%)	18 (39.13%)
Erythema nodosum	56 (19.85%)	12 (26.08%)
Infrequent skin rash	24 (8.51%)	3 (6.52%)
Gut involvement		
Persistent diarrhea	0 (0.0%)	27 (58.69%)
Hematochezia	0 (0.0%)	3 (6.52%)
Gastrointestinal occlusion	0 (0.0%)	1 (2.17)
Recurrent abdominal pain without peritonism	6 (2.12%)	15 (32.6%)
Recurrent abdominal pain with peritonism	0 (0.0%)	1 (2.17%)
Macroscopic inflammatory lesions at endoscopy**	0 (0.0%)	12 (26.08%)
Macroscopic vasculitic lesions at endoscopy**	0 (0.0%)	1 (2.17%)
Ocular involvement over time	26 (9.2%)	10 (21.7%)
Vascular involvement over time	11 (3.9%)	3 (6.45%)
CNS involvement over time	9 (3.2%)	1 (2.17%)

#, not provided in 22 cases; *in 60 cases HLA-B evaluation not performed; **Percentage referred to the 47 patients undergoing endoscopy; §, information provided in 222 (68%) cases; §§, information provided in 152 out of 220 cases with genital ulcers.

BDCAF, Behçet’s Disease Current Activity Form; CNS, central nervous system; F, females; HLA, human leukocyte antigen; M, males; SD, standard deviation.

**Table 2** presents the ORs and their corresponding 95% CrI, along with the probabilities of association between the specific variables assessed in this study and the development of intestinal involvement. The presence of minor aphthous ulcers with no major aphthosis was found to be protective against the development of intestinal involvement (OR = 0.47, 95% CrI: 0.22-0.98), with this assertion associated to a posterior probability of 97.98%. Conversely, the presence of major aphthous ulcers was associated with an increased predisposition to gut involvement (OR = 3.25, 95% CrI: 1.50-7.02), an assertion associated with a posterior probability of 99.7%.

**Table 2:** Results of Bayesian regression analyses evaluating the association between intestinal involvement (dependent variable) and the singularly assessed variables listed in the first column (independent variable).

Variables singularly assessed	OR (95% CrI)	P(β > 0)	P(β < 0)
Age at disease onset	1 (0.97-1.02)	41.15%	58.85%
Age at diagnosis	1 (0.98-1.03)	55.62%	44.38%
Sex (male)	0.6 (0.27-1.24)	9.4%	90.6%
Sex (female)	1.68 (0.79-3.7)	91.6%	8.4%
Body mass index, Kg	0.98 (0.95-1.01)	8.2%	91.8%
Smoking history	1.13 (0.44-2.7)	62.2%	27.8%
HLA-B51 positivity	2.05 (0.99-4.44)	97.2%	2.8%
HLA-B51 negativity	0.49 (0.22-1.04)	3.5%	96.5%
Positive family history	1.97 (0.6-5.8)	50.2%	49.8%
Negative family history	0.49 (0.22-1.04)	3.5%	96.5%
Initial BDCAF value#	1.25 (0.88-1.8)	88.8%	11.2%
Oral ulcers 1-2	0.43 (0.14-1.19)	5.4%	94.6%
Oral ulcers 3-5	1.73 (0.70-4.22)	89.5%	10.5%
Oral ulcers >5	1.11 (0.36-3.06)	58.67%	41.33%
Genital ulcers 1-2	1.04 (0.33-3.56)	51.5%	48.5%
Genital ulcers 3-5	1.52 (0.41-5.31)	74.5%	25.5%
Genital ulcers >5	0.01 (0-1.16)	3.42%	96.58%
Genital ulcers yes/no	1.63 (0.70-4.01)	86.5%	13.5%
Herpetiform aphthous ulcers	1.75 (0.21-10.8)	72.7%	27.3%
**Minor aphthous**	**0.47 (0.22-0.98)**	**2.02%**	**97.98%**
**Major aphthous**	**3.25 (1.50-7.02)**	**99.7%**	**0.3%**
Skin involvement	1.40 (0.67-3.09)	80.9%	19.1%
Pseudofolliculitis	1.07 (0.52-2.18)	56.5%	43.5%
Erithema nodosum	0.90 (0.41-2.29)	48.07%	51.93%
Other types of skin rash	0.17 (0.01-1.17)	4.05%	95.95%
Caucasic	2.45 (0.99-6.66)	97.42%	2.58%
Hispanic	0.81 (0.04-7.38)	48.0%	52.0%
Arab	0.5 (0.16-1.32)	9.22%	90.78%

#, Available in 78 patients.

Specifically, the table reports the odds ratio (OR, i.e., the exponentiated beta estimate), the 95% credibility interval (95% CrI), and the probabilities that the singularly assessed variable is either associated with P(β > 0) or protective against P(β < 0) intestinal involvement. All regressions are adjusted for the maximum disease duration and the use of immunosuppressive therapies. BDCAF, Behçet’s Disease Current Activity Form. In bold values that have a significant posterior probability (beta>97.5%).

Both HLA-B51 haplotype and Caucasian ethnicity showed a strong tendency towards a significant association with the development of intestinal involvement. In particular, HLA-B51 positivity showed an odds ratio of 2.05 (95% CrI: 0.99-4.44) for intestinal involvement, with a posterior probability of 97.2% to be associated to gastrointestinal involvement. Conversely, HLA-B51 negativity had an odds ratio of 0.49 (95% CrI: 0.22-1.04), with a posterior probability of 96.5% to be protective. Caucasian ethnicity showed an odds ratio of 2.45 (95% CrI: 0.99-6.66) for gut involvement, with a posterior probability of 97.42%.

**Table 3** presents the results from Bayesian regression models investigating the association between intestinal involvement and various combinations of mucocutaneous manifestations observed in the early stages of BD, after adjusting for follow-up duration and the use of cDMARDs or anti-TNF biologics. Specifically, the combination of oral major aphthous ulcerations and genital aphthosis was associated with an increased predisposition to intestinal involvement (OR = 2.77, 95% CrI: 1.14-6.58), with a posterior probability of 98.45%. Similarly, the co-occurrence of oral major aphthous ulcerations and pseudofolliculitis showed an increased predisposition to intestinal involvement (OR = 3.18, 95% CrI: 1.15-8.33), with a posterior probability of 98.72%. Furthermore, the triple association of oral major aphthous ulcerations, genital aphthosis, and pseudofolliculitis showed an even stronger predisposition to gut involvement (OR = 5.22, 95% CrI: 1.71-16.35), supported by a posterior probability of 99.77%. Conversely, the combination of pseudofolliculitis and cutaneous manifestations other than erythema nodosum was strongly associated with a protective effect against gut involvement (OR = 0.01, 95% CrI: 0.0-0.98), with a posterior probability of 97.55%.

**Table 3:** Results of Bayesian regression analyses evaluating the association between intestinal involvement (dependent variable) and different combinations of mucocutaneous manifestations of Behçet’s disease (independent variable).

Combination of at least two mucocutaneous features	OR (1-95%CI)	p (β>0)	p (β<0)
Minor oral aphtosis + Genital Aphthosis	0.70 (0.32-1.50)	19.05%	80.95%
Minor oral aphtosis + Pseudofolliculitis	0.45 (0.15-1.21)	6.05%	93.95%
Minor oral aphtosis + Erythema nodosum	1.06 (0.34-2.99)	56.62%	43.38%
Minor oral aphtosis + Other types of skin rash	0.01 (0.0-1.06)	2.63%	97.37%
**Major oral aphthous ulcerations + Genital Aphthosis**	**2.77 (1.14-6.58)**	**98.45%**	**1.55%**
**Major oral aphthous ulcerations + Pseudofolliculitis**	**3.18 (1.15-8.33)**	**98.72%**	**0.18%**
Major oral aphthous ulcerations + Erythema nodosum	2.37 (0.66-7.77)	92.0%	8.0%
Genital Aphthosis + Pseudofolliculitis	1.52 (0.74-3.10)	88.2%	11.8%
Genital Aphthosis + Erythema nodosum	1.02 (0.37-2.52)	52.57%	47.48%
Genital Aphthosis + Infrequent skin rash	0.23 (0.01-1.63)	9.12%	90.88%
Pseudofolliculitis + Erythema nodosum	0.33 (0.05-1.40)	7.82%	92.18%
**Pseudofolliculitis + Infrequent skin rash**	**0.01 (0.0-0.98)**	**2.45%**	**97.55%**
Minor oral aphtosis + Genital Aphthosis + Pseudofolliculitis	0.47 (0.13-1.45)	9.77%	90.23%
Minor oral aphtosis + Genital Aphthosis + Erythema nodosum	1.63 (0.49-4.97)	80.82%	19.18%
**Major oral aphthous ulcerations + Genital Aphthosis + Pseudofolliculitis**	**5.22 (1.71-16.35)**	**99.77%**	**0.33%**
Genital Aphthosis + Pseudofolliculitis + Erythema nodosum	0.44 (0.06-1.94)	15.42%	84.57%
Minor oral aphtosis + Genital Aphthosis + Pseudofolliculitis + Erythema nodosum	0.32 (0.01-2.43)	17.57%	82.43%

The table reports the odds ratio (OR, i.e., the exponentiated beta estimate), the 95% credibility interval (95% CrI), and the probabilities that the mucocutaneous combinations are either associated with P(β > 0) or protective against P(β < 0) intestinal involvement. All regressions are adjusted for the maximum disease duration and the use of immunosuppressive therapies. Only combinations of mucocutaneous manifestations observed in at least 10 patients were included in the statistical analysis. In bold values that have a significant posterior probability (beta>97.5%).

**Figure 1:**
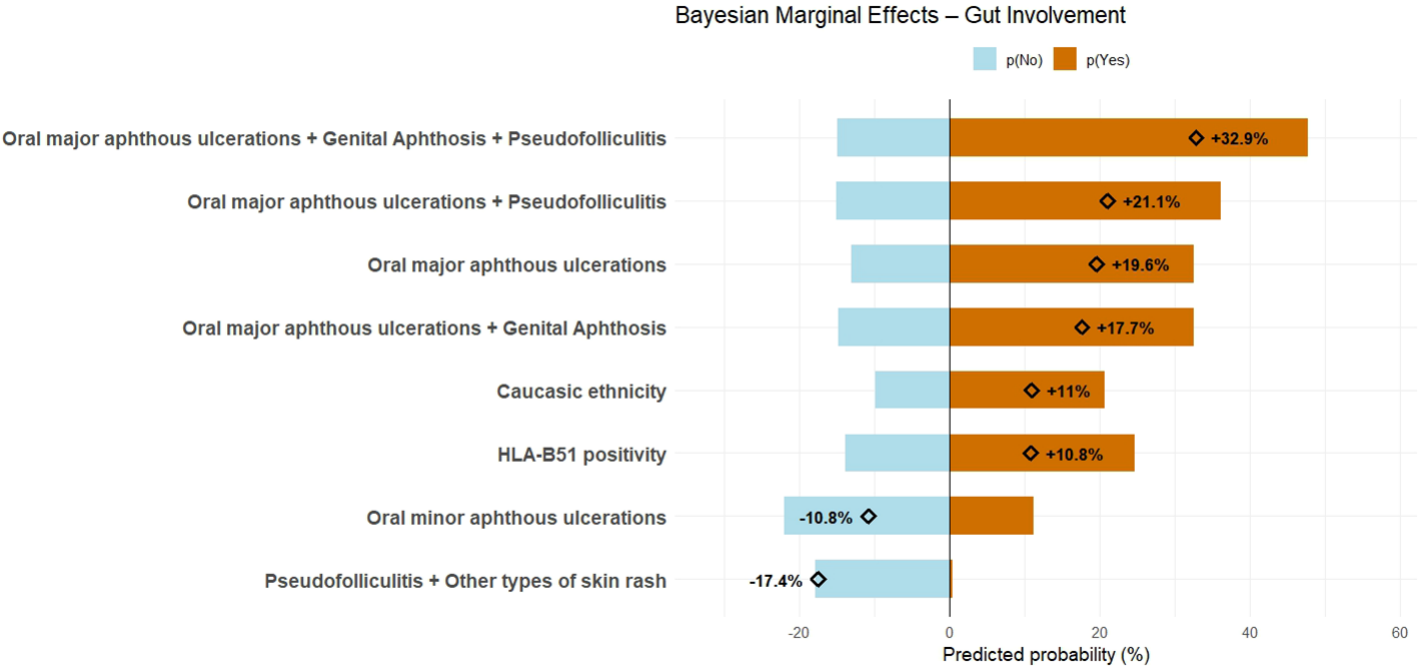
Balance plot illustrates the decreased (in blue, to the left) and increased (in orange, to the right) risk of developing intestinal involvement in patients who initially presented with mucocutaneous involvement only, based on the presence or absence of variables found to be significantly associated with intestinal involvement. For each variable, the marginal effect is reported as the absolute difference in predicted probability of intestinal involvement between patients with and without the given variable.

**Figure 1** displays a balance plot that illustrates the expected probabilities of gut involvement, conditional on the presence or absence of each variable significantly associated with intestinal inflammatory involvement. This plot also shows the marginal effects, which quantify the change in probability associated with the presence versus absence of each variable.

## Discussion

Intestinal involvement in BD patients, occurring in about 10-19% of cases (14), may significantly impair quality of life and potentially lead to serious and life-threatening complications (15). This can occur even in individuals initially presenting with a mild clinical picture and no major organ involvement in the early stage of the disease.

Independent factors associated to intestinal involvement in BD include male gender, higher disease activity, elevated inflammatory markers, and low haemoglobin (16). However, the present study aimed to identify factors associated with the development of intestinal involvement in the specific subset of patients with no major organ manifestations at disease onset. Particular attention was given to potential associations with mucocutaneous clinical features and patients’ demographic characteristics. The first prominent association observed was a higher frequency of ocular involvement among patients who also developed intestinal manifestations, suggesting a mutual predisposition for the co-occurrence of ocular and intestinal involvement in BD. This assertion, supported with 99.3% posterior probability at the current Bayesian analysis, stands in contrast to earlier findings reporting a lack of overlap between ocular and intestinal involvement in BD (14). However, this aforementioned finding was reported in a general cohort of BD Chinese patients, while the present study specifically investigated patients with mucocutaneous manifestations as their sole clinical presentation at disease onset. Also, the frequency of intestinal involvement in BD varies widely across populations, while patients from East Asian countries remain underrepresented in the AIDA registry, at current. In contrast, Hispanic, Arab, and Caucasian ethnicities are more prominently represented. When evaluating the risk of developing intestinal involvement among the most represented ethnic groups in this cohort, Caucasian ethnicity showed a trend toward a significant association with intestinal manifestations. Indeed, although the conventional threshold for statistical significance was not entirely satisfied, the association was nonetheless supported, with a posterior Bayesian probability of 97.42%.

Based on the marginal estimates obtained in the present study, suffering from exclusively minor aphthosis reduces the probability of developing gastrointestinal involvement by approximately 10%, whereas the presence of major aphthosis increases this probability by nearly 20%. The combination of major aphthosis and pseudofolliculitis further increases the risk to 21%, while patients presenting with major aphthosis, pseudofolliculitis, and genital aphthosis show an estimated 32% increase in the probability of developing gastrointestinal involvement. The association between major oral aphthosis and genital aphthosis alone also remains significantly correlated with intestinal inflammation, with an increased risk of approximately 17%. Therefore, major oral aphthosis represents the most robust predictor of intestinal involvement, as it consistently appears as the shared component in all statistically significant associations related to mucocutaneous manifestations. Despite the wide 95% CrI associated with the limited number of affected patients, the association of pseudofolliculitis with cutaneous manifestations distinct from erythema nodosum also appears to confer a protective effect against the development of intestinal involvement.

Noteworthy, current studies show that HLA-B51 is linked to a decreased prevalence of gastrointestinal involvement in BD (17). Interestingly, although not statistically significant, the present study shows a trend toward an association between HLA-B51 positivity and intestinal involvement in mucocutaneous-onset BD. Notably, the probability of an association between HLA-B51 positivity and the subsequent development of intestinal involvement is 97.2%, with a credibility interval approaching statistical significance. This discrepancy may be partly explained by the different ethnicities included in the previous studies, as they were predominantly conducted in Japanese and Korean cohorts (18–21). Alternatively, it might reflect a different role of HLA-B51 in the subgroups of BD patients with isolated mucocutaneous involvement in the early stages of the disease.

A positive family history is frequently observed in paediatric BD patients, and within the paediatric setting, an increased prevalence of gastrointestinal involvement has also been documented (22). In this study, a trend emerged linking a positive family history of BD to an increased risk of intestinal involvement in patients with an exclusively mucocutaneous disease onset. Specifically, the estimated probability of developing intestinal involvement increases by nearly 11% in patients with a positive family history compared to those without a familial background of BD.

Some study limitations should be acknowledged. Although the overall sample size was adequate, stratification by clinical features reduced the effective size of each subgroup. Using Bayesian statistics helped address this limitation more effectively than a traditional frequentist approach. As with all multicenter registry studies, variability in data collection is inevitable; however, internationality also reduces referral bias and enhances the representativeness of the study population. Intestinal involvement in this cohort was heterogeneous, ranging from recurrent abdominal pain with elevated faecal calprotectin to endoscopically confirmed mucosal lesions. For this reason, intestinal involvement was uniformly defined as the presence of inflammation and/or ulceration in the gastrointestinal tract, and all findings of the present study should be interpreted in light of this definition. The pathergy test was excluded from the analysis due to its inconsistent execution across centers, which limits its reliability (23–25). Also, the stratification of oral ulcers according to discrete numerical categories of lesion count (1-2, 3-5, >5) was not based on a validated classification system but was pragmatically defined according to the distribution of cases in our cohort, in order to avoid very small subgroups and preserve analytical interpretability. Finally, the precise time ranging between BD onset e the occurrence of gastrointestinal involvement was not reported in most of the patients and this information was not provided.

In conclusion, this study identifies major aphthosis as the strongest factor associated with the developing of intestinal involvement among BD patients initially presenting with exclusively mucocutaneous manifestations. The coexistence of major aphthosis with pseudofolliculitis, especially when genital aphthosis is also present, further increases the likelihood of intestinal involvement. Conversely, the presence of minor aphthosis alone appears to reduce the probability of developing intestinal involvement, as does pseudofolliculitis when occurring alongside cutaneous manifestations other than erythema nodosum. Finally, intestinal involvement is associated with the development of ocular inflammation, suggesting that in patients initially presenting with isolated mucocutaneous manifestations, the emergence of one may be concomitant with or predictive of the other.

## Data Availability

The raw data supporting the conclusions of this article will be made available by the authors, without undue reservation.
